# A home and community-based multicomponent physical activity intervention for older adults

**DOI:** 10.1093/geroni/igaf122.3472

**Published:** 2025-12-31

**Authors:** Jing Chen, Nan Hua, Qin Zhang

**Affiliations:** The First Affiliated Hospital, Zhejiang University School of Medicine, Hangzhou, Zhejiang, China (People’s Republic); The First Affiliated Hospital, Zhejiang University School of Medicine, Hangzhou, Zhejiang, China (People’s Republic); The First Affiliated Hospital, Zhejiang University School of Medicine, Hangzhou, Zhejiang, China (People’s Republic)

## Abstract

Feasible physical activity (PA) interventions to promote multicomponent PA among community-dwelling older adults in China are limited. We aimed to develop and test the implementation feasibility of a multicomponent PA intervention for community-dwelling older adults. The intervention was developed by a multidisciplinary working group based on behavior change theories and techniques. A 12-week feasibility study was conducted in a community in Hangzhou, China. Feasibility was tested by Bowen’s framework for assessing acceptability, demand, implementation, practicality, adaptation, integration, expansion, and limited efficacy of the PA intervention program. The PA intervention combined face-to-face group exercise (twice a week) in a community center and home-based exercise assisted by a WeChat applet or exercise manuals (at least once a week) with a weekly goal of engaging in multicomponent exercise at least three days per week. Overall, 19 participants (95%) completed the intervention (mean age of 77 years) with 12 participants using the applet and seven using the exercise manuals for home-based exercises. The intervention satisfied all Bowen’s feasibility criteria. In the third month, 93% of the applet users and 79% of the non-applet users adhered to weekly exercise goals. Significant improvements were observed in grip strength (+1.4 kg, 95% CI 0.1 to 2.7) and chair stand test (-3.5 seconds, 95% CI -4.9 to -2.2). The findings suggested that the PA intervention program was feasible in real-world settings, demonstrating its potential for promoting multicomponent PA among community-dwelling older adults. A large-scale randomized controlled trial is needed to test the long-term effectiveness.

